# Polarization vision in terrestrial hermit crabs

**DOI:** 10.1007/s00359-023-01631-z

**Published:** 2023-04-12

**Authors:** Martin J. How, Alasdair Robertson, Samuel P. Smithers, David Wilby

**Affiliations:** 1 School of Biological Sciences, University of Bristol, Bristol, UK; 2 Department of Psychology, Northeastern University, Boston, MA USA; 3 Research Software Engineering Team, Department of Computer Science, University of Sheffield, Sheffield, UK

**Keywords:** Polarization, Hermit crab, Visual ecology, Crustacean, Vision

## Abstract

Polarization vision is used by a wide range of animals for navigating, orienting, and detecting objects or areas of interest. Shallow marine and semi-terrestrial crustaceans are particularly well known for their abilities to detect predator-like or conspecific-like objects based on their polarization properties. On land, some terrestrial invertebrates use polarization vision for detecting suitable habitats, oviposition sites or conspecifics, but examples of threat detection in the polarization domain are less well known. To test whether this also applies to crustaceans that have evolved to occupy terrestrial habitats, we determined the sensitivity of two species of land and one species of marine hermit crab to predator-like visual stimuli varying in the degree of polarization. All three species showed an ability to detect these cues based on polarization contrasts alone. One terrestrial species, *Coenobita rugosus*, showed an increased sensitivity to objects with a higher degree of polarization than the background. This is the inverse of most animals studied to date, suggesting that the ecological drivers for polarization vision may be different in the terrestrial environment.

**Supplementary Information:**

The online version contains supplementary material available at 10.1007/s00359-023-01631-z.

## Introduction

The use of the polarization of light for object detection has been demonstrated in a range of animal groups, including cephalopods, crustaceans and insects (How et al. 2012; Temple et al. 2012; Drerup and How 2021; Shashar and Cronin 1996; Cronin et al. 2002; Kelber et al. 2001). These ‘small-field’ tasks require the detection or identification of objects such as predators, food, or conspecifics in small parts of the visual field, roughly analogous to the colour vision systems that many animals use to detect objects based on chromatic contrasts. Small-field tasks differ from wide-field tasks, such as navigation and orientation using the pattern of polarization in the sky (Wehner 1976).

Object-based polarization vision is particularly widespread in aquatic and semi-aquatic species, suggesting that these habitats are well-suited for the detection of objects using contrasts in polarization (Marshall et al. 2019). One explanation for this is that these environments offer predictably polarized backgrounds. For example, in clear water, the background illumination produced by the scattering of down-welling light from the sun and sky is generally polarized (up to 50–70%) and is predominantly oriented along the horizontal axis (Cronin and Shashar 2001). Similarly, on intertidal mudflats the damp substrate reflects light from the sun and sky also with a predominantly horizontal angle of polarization (How et al. 2015). In terrestrial and semi-terrestrial habitats, the blue sky also provides a stable and predictable backdrop of polarization, with angle and degree relating to the position of the sun (Wehner 1976; Homberg 2004). These predictably polarized backgrounds provide an ideal scene against-which to spot unpolarized contrasts associated with predators, food, or conspecifics (How et al. 2015). A second explanation for why polarization vision may be so prevalent underwater is that, particularly in shallow aquatic environments, this sensory modality may help to mitigate challenges associated with dynamic lighting (Vincent Venebles et al. 2022), a theory first proposed by Maximov (2000) to explain the evolution of colour (rather than polarization) vision underwater.

In many terrestrial environments, polarization may be a less reliable source of information for threat detection than colour, due to high levels of polarization ‘noise’ generated by background objects such as vegetation. On land, the large difference in refractive index between air and natural objects is often responsible for complex polarization cues across visual scenes. This differs from underwater, where the difference in refractive index between objects and water is much lower, leading to much reduced levels of polarized reflections (Marshall et al. 2019). Despite this, there are terrestrial species that have evolved small-field polarization vision that mediate specific behavioural tasks. For example, some butterflies combine polarization and spectral information to help locate appropriate vegetation for oviposition (Kelber et al. 2001; Blake et al. 2020; Nagaya et al. 2021) and many insect species use small-field polarization cues for locating aquatic habitats or bloodmeal hosts (Schwind 1984; Kriska et al. 1998; Horváth et al. 2008; Mathejczyk and Wernet 2017; Heinloth et al. 2018; Meglič et al. 2019; Yadav and Shein-Idelson 2021).

The apparent rarity of polarization vision systems for threat detection in terrestrial environments opens the question of whether marine species that have evolved to occupy terrestrial habitats would lose any survival advantage conferred by their polarization vision, and therefore lose the ability to detect predators using the polarization of light through the process of genetic drift. A candidate group of animals for investigating this question is the superfamily of hermit crabs, the Paguroidea (Latreille 1802).

There are some 800 species of hermit crab, most of which occupy marine or intertidal habitats (Hazlett 1981). One family, the Coenobitidae (Dana 1851), have evolved to exploit terrestrial ecosystems (Hartnoll 1988) and display a range of adaptations for life away from water (Burggren and McMahon 1988). These species spend their entire lives in terrestrial habitats, returning to the ocean only to release larvae (Nio et al. 2019). Two species of terrestrial hermit crab are abundant at our study site on the island of Mahé, Seychelles. *Coenobita rugosus* (Milne Edwards 1837) tend to occupy forest understory from the top of the shoreline to approximately 100 m inshore, and *Coenobita brevimanus* (Dana 1852) occupy more inland forest habitats > 50 m inshore (Hsu et al. 2018). Whether or not these species make use of polarization vision is unknown, but it is likely that—much like their marine counterparts (Kerz 1950)—ancestral marine hermit crabs would have had this sensory capability.

To determine whether *C. rugosus* and *C. brevimanus* make use of the polarization of light, we determined their sensitivity to predator-like visual cues presented only in contrasts of polarization using an established playback assay (How et al. 2012; Shragai et al. 2017; Wilby et al. 2018) and compared this to the polarization responses of a fully marine species, *Pagurus bernhardus* (Linnaeus 1758). To interpret our findings, we analysed the distribution of polarization cues in the visual habitats of the two terrestrial species by collecting footage with a polarization camera on the island of Mahé, Seychelles.

## Materials and methods

### Animal collection

Terrestrial hermit crabs (*C. rugosus* and *C. brevimanus*) were collected at Anse Petit Police (4°48′12″S; 55°31′05″E) and Anse Marie-Louise (4°47′24″S; 55°31′50″E), Mahé Island, Seychelles and housed for < 48 h in a terrarium with damp plant detritus. Animals were maintained in natural light cycles and fed ad libitum on fruit scraps (coconut, papaya and mango). Marine hermit crabs (*P. bernhardus*) were collected from intertidal rockpools at Looe, Cornwall, UK (50°20′57″N; 4°27′01″W), housed for < 2 weeks in an aquarium with artificial seawater at salt concentrations of 35ppt, and fed twice weekly with defrosted shrimp. All animals were returned to the site of collection after experimentation.

### Experimental procedure

**Fig. 1:**
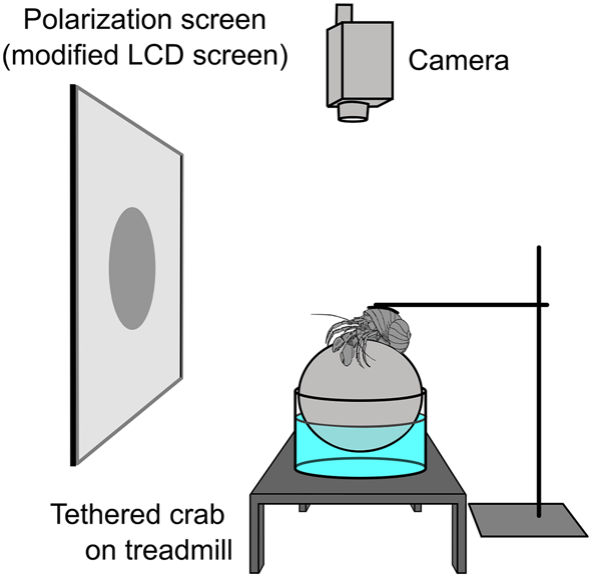
Experimental apparatus for presenting polarized expanding cues to tethered hermit crabs. For terrestrial species, the crab was tethered above a Styrofoam sphere floating in a cup of water. For marine species the crab was tethered at the bottom of a small seawater aquarium

The response of hermit crabs to visual stimuli was tested using an approach similar to previous studies on other crab species (How et al. 2012, 2015; Shragai et al. 2017; Wilby et al. 2018; Smithers et al. 2019). Briefly, animals were tethered above a treadmill (for terrestrial species) or underwater over a slippery surface (for the marine species) so that they faced a display screen on which moving stimuli could be presented (Fig. 1). For terrestrial crabs, the stimulus screen consisted of a field-portable patterned-vertical-alignment type liquid crystal display (LCD) screen (1905fp, Dell Technologies, Round Rock, USA) with the front-most polarizing filter replaced with a removable sheet of polaroid filter (see Foster et al. 2018 for details). For the marine species, a lab-based version of the screen was constructed, consisting of 1) an LCD panel (1905fp, same as above) with the outer most polaroid filter removed and dissembled from the panel housing to allow illumination from 2) a digital projector (CP-WX3030WN, Hitachi Ltd., Tokyo, Japan) (see Smithers et al. 2019 for further details).

A visual stimulus simulating the looming movement of an approaching predator was created by animating an expanding disc on the display. The disc expanded around a central point with a geometric expansion profile over a period of 3 s for terrestrial hermits and 10 s for *P. bernhardus*, from a visual angle of 0° to approximately 19° (12 cm disc diameter viewed from ~ 35 cm), followed by a 2 s period at maximum size before disappearing. By modifying the RGB (R = G = B) values of the pixels of the background and foreground of the stimulus, we could systematically adjust either the intensity contrast (presented with the removable polarizer in place or on the digital projector screen) or the polarization contrast (presented with the removable polarizer removed or on the polarization screen) of the expanding disc. Polarization and intensity properties of the screen were measured using a spectrophotometer (Flame, Ocean Optics, Orlando, USA) coupled to a Glan Thompson linear polarizer (GTH10M-B, Thorlabs, Newton, USA).

After acclimatising to the experimental arena for 3 min, animals were presented with a randomised series of expanding disc stimuli varying in contrast with the background, with time intervals of 1–3 min. Animals only saw one series of stimuli in either intensity or polarization and so each series of contrasts provides an independent set of results. For intensity-based (I) stimuli, the background was set to near the mid-point of the radiance levels measured across the greyscale range, which corresponded to 8 bit RGB values of [158, 158, 158]. Expanding disc stimuli varied from this in both the negative (darker) and positive (lighter) weber contrast scale, defined as

$${\text{Weber}}\, {\text{contrast}} = \frac{{I_{{{\text{object}}}} - I_{{{\text{background}}}} }}{{I_{{{\text{background}}}} }}.$$

For polarization-based stimuli, the background was set to 50% polarized in the horizontal plane, with stimuli differing in degree of polarization (DoP) in the negative (less polarized) and positive (more polarized) scale (for details on all pixel values, weber contrast and DoP of stimuli, see Supplementary Table 1). Crab responses were recorded with a digital video camera filming from above, and stimulus timing was passed to the video stream both by audio signals conveyed through the microphone port (inaudible to the outside world) and via a red flashing light-emitting-diode (LED) placed outside the field of view of the hermit crab. The LED emitted no wavelengths of light below 600 nm and so falls outside the main spectral sensitivity range of the animals.

### Behavioural data analysis

Responses of hermit crabs to the visual stimuli were determined using a blind-scoring technique, and for terrestrial crabs, the timing of onset of three different behaviours was determined relative to the stimulus. These behaviours were: *stop*, the animal stopped its previous behaviour and became stationary; *retreat*, the animal pulled itself partially or fully into its shell refuge; and *walk*, the animal began walking on the treadmill. A crab was said to have responded to a stimulus if it exhibited any of these behaviours. A small number of trials were excluded if the animal performed a full retreat before the onset of the visual stimulus, a behaviour presumed to be associated with hydration.

Contrast response curves were modelled (where possible) with sigmoid functions using the sigm_fit script (Pavao 2017) in Matlab (r2021, Mathworks, Natick, USA) and habituation was modelled by fitting a line to response probability as a function of stimulus order using Matlab’s ‘polyfit’ function. Data for intensity responses of *P. bernhardus* were not collected over a suitable range of contrasts for comparison with terrestrial species, so the graph is not included for this species.

For statistical analysis, we used mixed effects binary logistic regression, fitted using the lme4 package (Bates et al. 2015) in R (R Core Team 2022). We used the DHARMa package (Hartig 2022) to run residual diagnostics that are often overlooked in logistic regression. The binary response variable was crab response. Stimulus contrast (i.e. intensity or polarization contrast) and presentation order were included as fixed effects, the latter was included to test for habituation. Crab ID was included as a random effect to control for repeated measures. When fitting the full model for the intensity experiment with *C. rugosus* a quadratic effect was included to control for an irregular pattern in the residuals. To test for a significant effect of stimulus contrast and presentation order we used a likelihood ratio test (LRT) to compare the full model with the same model but with the effect of interest removed.

### Polarization camera recording

To investigate the polarization environment of *C. rugosus* and *C. brevimanus*, a monochromatic polarization camera (Triton TRI050S-PC, LucidVisionLabs, Richmond, Canada) was used to take snapshots from the location at which animals were collected for study. The polarization camera collects information via subpixels etched with polarization filters oriented at 0°, 45°, 90° and 135° and the local degree and angle of polarization values for each super-pixel were then calculated using stokes equations and displayed in false-colour images (see Foster et al. 2018 and supplementary information for details).

## Results

### Behavioural experiments

**Fig. 2:**
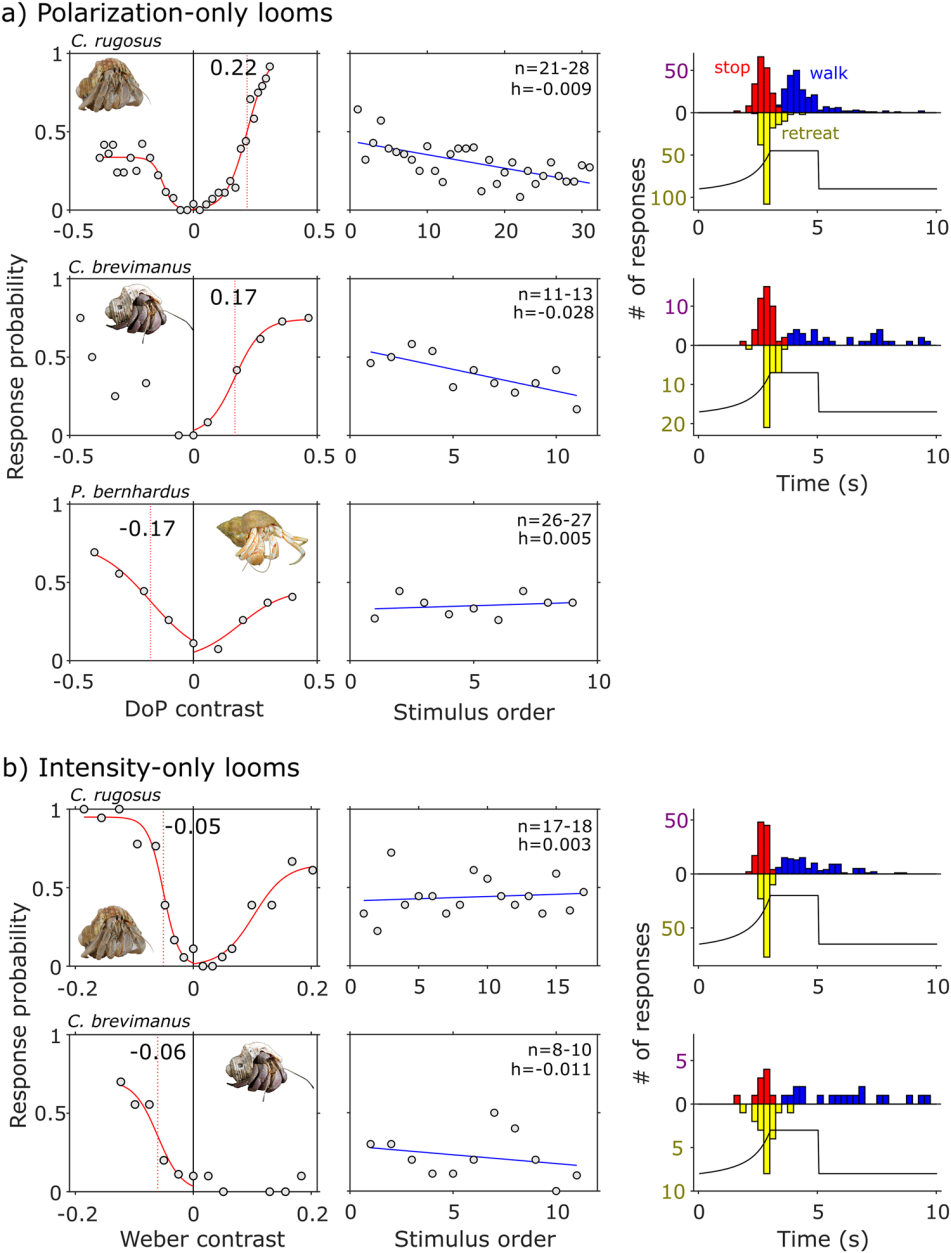
Responses of hermit crab species to visual stimuli presented in **a** polarization contrast only (DoP contrast is calculated as stimulus-background DoP values), and **b** intensity contrast only (negative and positive values equate to darker and lighter stimuli than the background respectively). Left panels: response as a function of stimulus contrast. Solid red lines show (where possible) fitted sigmoid curves. Vertical dotted red lines show steepest point of sigmoid slope. Middle panels: response as a function of stimulus order. Blue lines show lines of best fit. Note the differences in x-axis scale. ‘h’ = habituation rate, defined as the change in response probability per trial. Right panels: timing and frequency of ‘*stop*’ (red), ‘*retreat*’ (yellow), and ‘*walk*’ (blue) behaviours relative to loom expansion (black line). Note that ‘retreat’ behaviour is scored as positive values below the x-axis. Sample sizes encompass data arranged by contrast and by order of presentation. Inset photos by M How and H Hillewaert

Both the marine *P. bernhardus* and the terrestrial *C. rugosus* showed evidence of good polarization vision responding reliably to predator-like stimuli varying only in polarization contrast (Fig. 2a, left; mixed effects binary logistic regression- effect of polarization contrast for *P. bernhardus*: χ^2^_(1)_ = 12.31, *p* < 0.001; effect of polarization contrast for *C. rugosus*: χ^2^_(1)_ = 32.16, *p* < 0.001). Both species exhibited an asymmetric response curve indicating that the effect of polarization contrast is driven by both the size and polarity of the contrast. The terrestrial crab *C. brevimanus* also exhibited evidence of polarization vision (Fig. 2a, left), although polarization contrast was not found to be statistically significant (χ^2^_(1)_ = 2.41, *p* = 0.12). However, as is evident from Fig. 2a, we argue that this result is not due to a lack of response to polarization contrasts. Unlike for the other two species, the response curve for *C. brevimanus* is relatively symmetrical on either side of the control suggesting that contrast polarity is less important for this species. Given the reduced effect of contrast polarity for *C. brevimanus*, it is possible that the smaller sample size meant that the model did not have enough power to detect the overall effect of polarization contrast. This suggestion is supported by the fact that the effect of polarization contrast is highly significant when we fit separate models for positive (χ^2^_(1)_ = 28.36, *p* < 0.001) and negative (χ^2^_(1)_ = 25.4, *p* < 0.001) contrasts. Half-maximum values extracted from fitted sigmoidal curves indicate threshold response values of DoP between 0.17 and 0.22 for all species. Interestingly, the terrestrial *C. rugosus* showed a stronger response to positive polarization contrasts (stimuli with a higher DoP than the background), while the marine species showed a stronger response to stimuli with a lower polarization contrast than the background.

Both terrestrial species responded more typically to intensity-only stimuli (effect of intensity contrast for *C. rugosus*: χ^2^_(1)_ = 81.53, *p* < 0.001; effect of intensity contrast for *C. brevimanus*: χ^2^_(1)_ = 30.4, *p* < 0.001), showing the strongest response to darker expanding discs than the background (Fig. 2b, left). The types of behaviour and their timing relative to stimulus onset was similar between species and across both polarized and intensity-based stimuli (Fig. 2a,b, right). In the polarization experiment both terrestrial species displayed a significant amount of habituation (Fig. 2a, middle; effect of order for *C.* rugosus: χ^2^_(1)_ = 25.46, *p* < 0.001; effect of order for *C. brevimanus*: χ^2^_(1)_ = 4.93, *p* = 0.026), while *P. bernhardus* did not (effect of order for *P. bernhardus*: χ^2^_(1)_ = 0.79, *p* = 0.375) (Fig. 2a, middle). *Coenobita rugosus* also showed signs of habituation in the intensity experiment (effect of order for *C. rugosus*: χ^2^_(1)_ = 5.43, *p* = 0.02), but *C. brevimanus* did not (effect of order for *C. brevimanus*: χ^2^_(1)_ = 1.6, *p* = 0.207).

### Terrestrial polarization

**Fig. 3:**
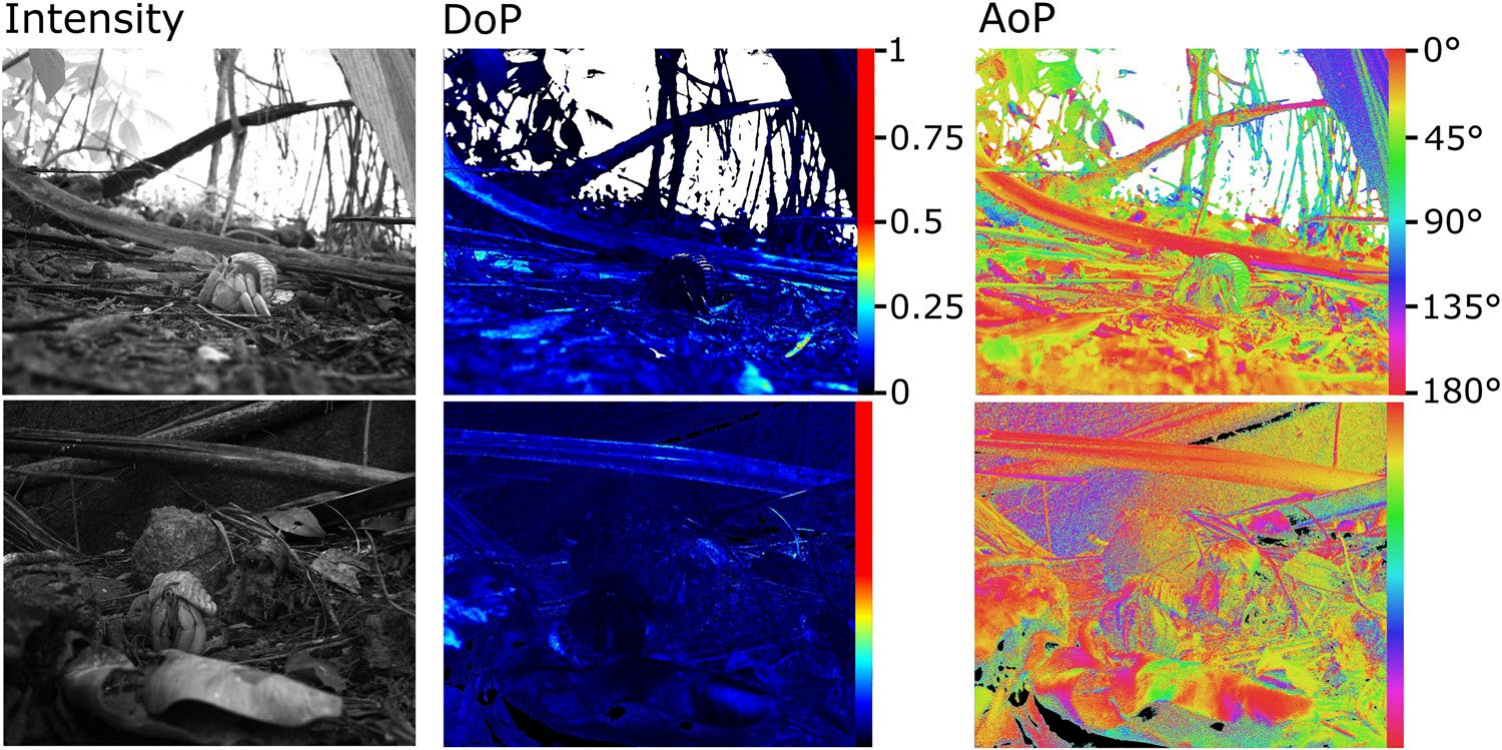
Polarimetry from the terrestrial habitats of *C. rugosus* (top) and *C. brevimanus* (bottom). Left: Intensity image. Middle: Degree of polarization (DoP) false-colour image (scale along right of panel). 0 = unpolarized, 1 = fully polarized. Right: Angle of polarization (AoP) false-colour image; 0° is horizontal. Over- and under-exposed pixels are assigned white and black respectively in the DoP and AoP images

Polarization images taken from a crab’s perspective at the site of animal collection indicates relatively low levels of DoP (Fig. 3, middle). These locations consisted of leaf litter, soil and sand under a canopy of palms and deciduous trees. The locations were also characteristically dry at the time of measurement.

## Discussion

Our results demonstrate that terrestrial hermit crabs can use polarization contrasts in the context of threat detection, despite evolving to occupy terrestrial habitats with very different polarization content to marine and intertidal locations. Whether this is because polarization vision conveys a fitness benefit to these species, or whether this sensory capacity is an evolutionary hang-over from their previous marine adaptations remains to be demonstrated.

One result of note is that the terrestrial species exhibited standard responses to intensity stimuli, showing stronger responses to dark objects viewed against light backgrounds than vice versa. This behavioural filter is likely due to silhouetted predators appearing darker than the background and can similarly be found in many other species (Layne 1998; Santer et al. 2005; Yilmaz and Meister 2013; Temizer et al. 2015; Smithers et al. 2019). However, the terrestrial crab *C. rugosus* differed from the marine species, and indeed other crustaceans (Layne 1998; Smithers et al. 2019), by responding more strongly to stimuli with higher DoP than the background (Fig. 2a, left). In other contexts, such as underwater or in semi-terrestrial mudflats, animals benefit from polarization vision by being able to detect objects with low levels of polarization (be they conspecifics or predators) viewed against a uniformly polarized background of reflected or scattered light (How et al. 2015). Perhaps the visual ecology of terrestrial hermit crabs is different and salient objects in the environment tend to be more polarized than the unpolarized background, causing behavioural filters to favour responding to cues that are more polarized than the background. This possibility is supported by the polarimetry (Fig. 3) which shows very low levels of polarization reflected from the dry understory of inland forest habitats occupied by these species. How these polarization and intensity channels (and indeed any chromatic input if present) of information are integrated in the optic lobes of the hermit crab is unknown, but could be tested using a similar approach to Smithers et al (2019).

Another possibility is that polarization vision serves alternative ‘small field’ visual tasks such as the location of damp understory refuges or fresh water sources needed during dry conditions (Mathejczyk and Wernet 2017). Or alternatively, polarization vision may play a role in navigation when moving towards or away from shore during reproductive behaviour (Vannini and Chelazzi 1981). Future research should target the ecological function of polarization vision in these species.

### Supplementary Information

Below is the link to the electronic supplementary material.

Supplementary file1 (DOCX 232 KB)

## Data Availability

All datasets and analysis code are available at the github repository https://github.com/EcologyOfVisionBristol/HermitCrabPolarizationVision2023.git.
