# Supplementary material for: Polarization vision in terrestrial hermit crabs
Source: J Comp Physiol A Neuroethol Sens Neural Behav Physiol. 2023 Apr 12;209(6):899–905. doi: 10.1007/s00359-023-01631-z (PMC10643299; doi:10.1007/s00359-023-01631-z)
Supplement: Supplementary file 1 — Supplementary file1 (DOCX 232 KB) [file 359_2023_1631_MOESM1_ESM.docx]

**Supplementary information**

**Table S1.** Background and stimulus pixel values and their corresponding degree of polarization or weber contrast.

| **Species** | **Stimulus type** | **Background** | **Stimulus**  **(pixel value** and *DoP or Weber contrast***)** |
| --- | --- | --- | --- |
| *C. rugosus* | Polarization | **158**  *0.50* | **98, 102, 106, 110, 114, 118, 122, 126, 130, 134, 138, 142, 146, 150, 154, 158, 162, 166, 170, 174, 178, 182, 186, 189, 192, 195, 198, 200, 202, 204, 206**  *0.80, 0.79, 0.77, 0.76, 0.74, 0.72, 0.71, 0.69, 0.67, 0.64, 0.62, 0.60, 0.57, 0.55, 0.52, 0.50, 0.47, 0.44, 0.41, 0.38, 0.35, 0.32, 0.29, 0.26, 0.24, 0.21, 0.19, 0.15, 0.17, 0.13, 0.12* |
| *C. brevimanus* | Polarization | **158**  *0.50* | **35, 84, 108, 129, 149, 158, 167, 184, 199, 215, 227**  *0.96, 0.85, 0.77, 0.67, 0.55, 0.50, 0.44, 0.30, 0.18, 0.09, 0.04* |
| *P. bernhardus* | Polarization | **154**  *0.52* | **67, 96, 120, 138, 154, 168, 181, 193, 205**  *0.92, 0.82, 0.72, 0.62, 0.52, 0.42, 0.32, 0.22, 0.12* |
| *C. rugosus* | Intensity | **127** | **115, 117, 119, 121, 123, 124, 125, 126, 127, 128, 129, 130, 131, 133, 135, 137, 139**  *-0.19, -0.16, -0.13, -0.09, -0.06, -0.05, -0.03, -0.02, 0, 0.02, 0.03, 0.05, 0.07, 0.10, 0.13, 0.17, 0.20* |
|  |  |  |  |
| *C. brevimanus* | Intensity | **158** | **148, 150, 152, 154, 156, 158, 160, 162, 168, 170, 172**  *-0.12, -0.10, -0.07, -0.05, -0.03, 0 0.03, 0.05, 0.13, 0.16, 0.18* |

**Polarization camera**

Polarization images were captured using a Triton GigE camera (TRI050S-PC, LucidVisionLabs, Richmond, Canada) equipped with a monochrome IMX264MZR polarization CMOS (complementary metal oxide semi-conductor) sensor (Sony, Tokyo, Japan). At the time of writing, many details of chip architecture and spectral sensitivity were available at the manufacturer’s website (<https://thinklucid.com>). The website lacks data on sensor linearity, so this was checked by imaging a greyscale standard and comparing sensor values with relative reflectance measurements collected with a spectrophotometer (HDX, OceanInsight, Orlando, USA) fitted with a bifurcated reflectance probe (R400-7-UV-VIS, OceanInsight) mounted in a 45° probe holder (RPH-1, OceanInsight) and illuminated with a broad-spectrum halogen light source (HL-2000-HP, OceanInsight). Reflectance measurements were normalised to the brightest greyscale measurement. This process confirmed that raw images captured by the camera had a strong linear sensitivity (Supp fig 1) and so no gamma correction steps were necessary for subsequent polarization calculations.


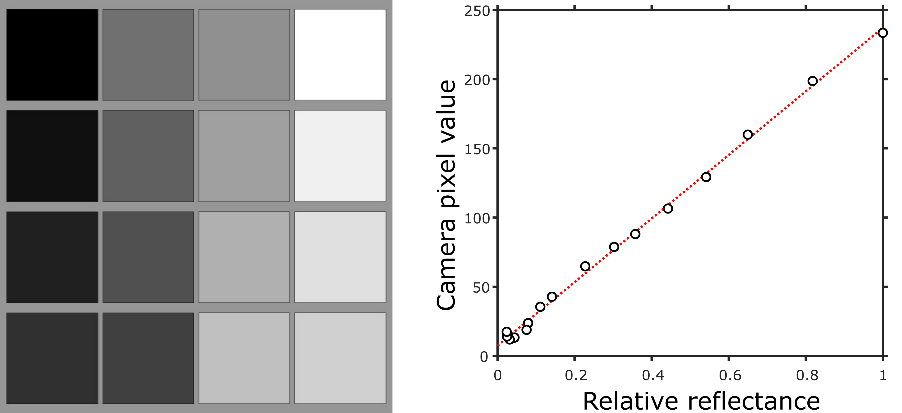


**Supplementary Figure 1. Linearity test for polarization camera.** Pixel values extracted from images of a printed greyscale standard (left) are plotted against relative reflectance values for each corresponding shade of grey. Red dotted line is the fitted line calculated using the least sum of squares method.

Briefly, each pixel on the camera CMOS array is composed of four adjacent sub-pixels, each overlaid with a nano-scale wire-grid acting as a polarization filter oriented at 0°, 45°, 90° or 135°. To extract the four polarization channels of information from the raw mosaic image, a simple demosaicking algorithm (illustrated in supplementary figure 2) was implemented in Matlab (r2022a, Mathworks, Natick, USA) using the following script.

**
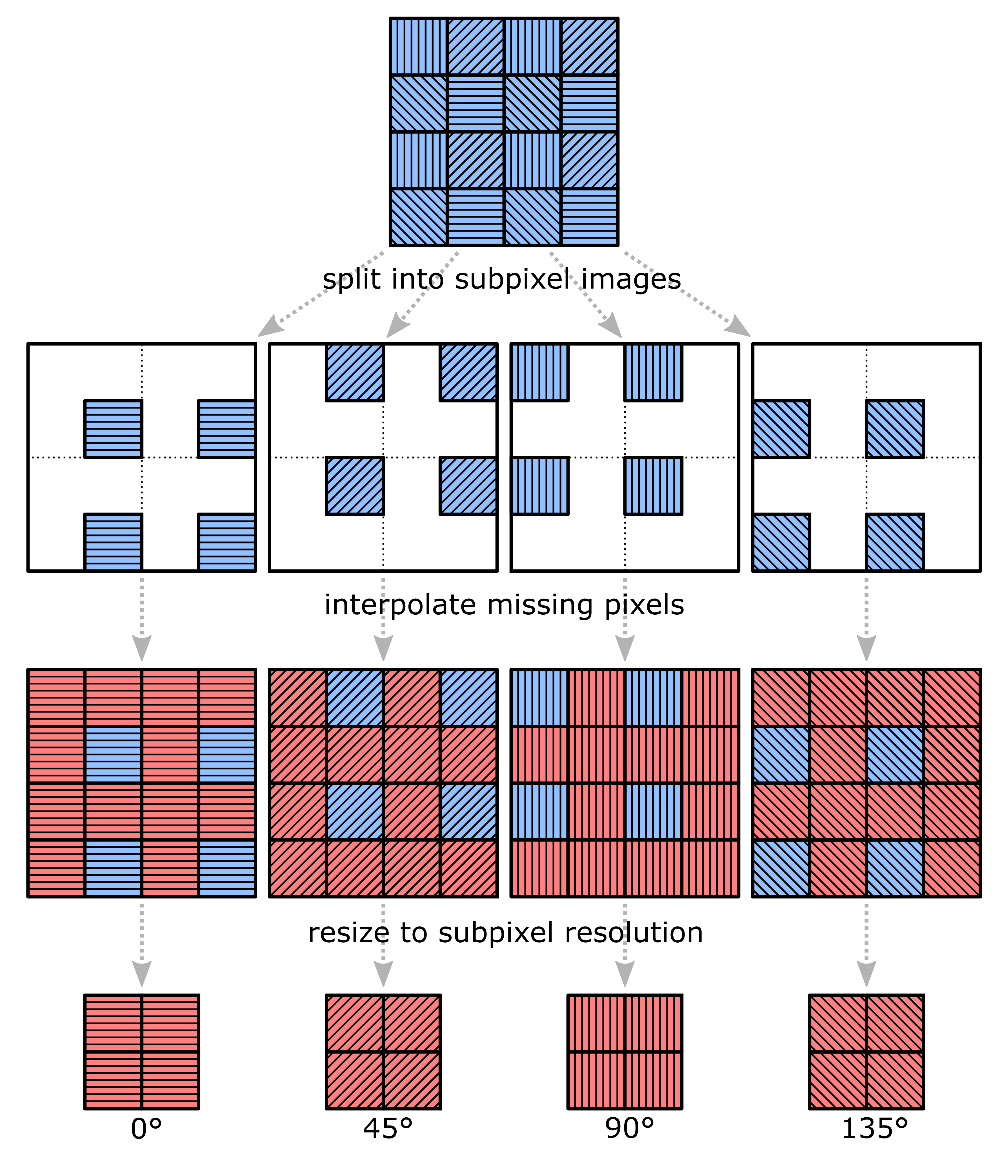
**

**Supplementary Figure 2. Method for demosaicking raw image frames from the polarization camera.** First, four copies of the mosaic image are created, each containing data from one subpixel class. Second, the empty subpixels are populated by data interpolated from the existing subpixels using the bicubic spline method. Third, the interpolated images are resized by 50% to produce a final set of images with subpixel resolution.

function [I0,I45,I90,I135] = demosaicPolCam(RawImage)

%Prepare meshgrids for interpolation step

[hei,wid,~] = size(RawImage);

[xx,yy] = meshgrid(1:2:wid,1:2:hei);

[xq,yq] = meshgrid(1:wid,1:hei);

for ii=1:4

if ii==1, y=1; x=1; end

if ii==2, y=1; x=2; end

if ii==3, y=2; x=2; end

if ii==4, y=2; x=1; end

%Extract the data channel from the mosaic image

im(:, :) = cast(RawImage(y:2:end, x:2:end,1), 'single');

%Reconstruct full-size image using 2d interpolation

im = interp2(xx+x-1,yy+y-1,im,xq,yq,'spline');

%Resize to original and confine scale to uint8 range

im = double(uint8(imresize(im,1/2)));

%Assign to output images for each channel

if ii==1, I0 = im; end

if ii==2, I45 = im; end

if ii==3, I90 = im; end

if ii==4, I135 = im; end

end

Once the four polarization images have been extracted from the mosaic image, any over or underexposed pixels were identified by indexing pixel values within 2% of the minimum (0) and maximum (255) 8-bit greyscale range. Then, the three stokes parameters were calculated pixel-by-pixel using the following script.

% Calculate Stokes parameters - S0, S1, S2.

S0 = I0 + I90;

S1 = I0 - I90;

S2 = I45 - I135;

Finally, the degree of polarization (DoP) and angle of polarization (AoP) was calculated as follows.

% Calculate DoP from Stokes parameters

DoP_tmp = S1.^2 + S2.^2;

DoP = (sqrt(DoP_tmp)) ./ S0;

% Calculate AoP from Stokes parameters

AOP = 0.5 .* atan2(S2, S1);

AOP = AOP + pi/2;

AOP = AOP .* 180/pi;

Pixel-by-pixel DoP and AoP matrices were then converted into false-colour RGB images by implementing a colour map and lookup routine. For DoP, the colour map extended from DoP of 0 to 0.5 by setting ‘dopThresh’ in the following function to [0 0.5]. For AoP the colour map extended across the full 0° to 180° range.

function rgb_out = deg2color(DoP, dopThresh, ind_under, ind_over)

%Function to assign false colour to DoP polarization matrix

%DoP – degree of polarization matrix

%dopThresh – minimum and maximum values for colour scale [min max]

%ind_under – index of any underexposed pixels to be discounted from calculations

%ind_over – index of any overexposed pixels to be discounted from calculations

%Compress DoP values to lie withing threshold range

DoP = (DoP-dolpThresh(1))./(dolpThresh(2)-dolpThresh(1));

DoP(DoP<0)=0;

DoP(DoP>1) = 1;

%Define false colour map

colscale = ...

[0 0 0; %Black

0 0 1; %Blue

0 1 1; %Cyan

1 1 0; %Yellow

1 0 0];%Red

%Map dop values to false colours in colour map

degrange = [0:1/(size(colscale,1)-1):1]';

for rgb=1:3

%Assign colours to output image

col = colscale(:,rgb);

imtemp = interp1(degrange,col,DoP);

%Assign underexposed pixels black and overexposed pixels white

imtemp(ind_under) = 0;

imtemp(ind_over) = 1;

rgb_out(:,:,rgb) = imtemp;

end

%Generate final rgb image

rgb_out = uint8(rgb_out.*255);

function [ aopRGB ] = aopToColor( AoP , ind_under, ind_over)

%aopToColor creates false colour AoP image

%First normalise to 1

AoP = AoP/180;

%Then create HSV image

aopHSV(:,:,1) = AoP;

aopHSV(:,:,2) = 0.8;

aopHSV(:,:,3) = 0.9;

%Then convert to RGB

aopRGB = hsv2rgb(aopHSV);

aopRGB = uint8(aopRGB*255);

%Finally, assign black to underexposed and white to overexposed pixels

for rgb=1:3

temp=aopRGB(:,:,rgb);

temp(ind_under) = 0;

temp(ind_over)=255;

aopRGB(:,:,rgb)=temp;

end
